# Dolutegravir Inhibits Proliferation and Motility of BT-20 Tumor Cells Through Inhibition of Human Endogenous Retrovirus Type K

**DOI:** 10.7759/cureus.26525

**Published:** 2022-07-03

**Authors:** Jiayi Li, John Lin, John R Lin, Mason Farris, Lauren Robbins, Leo Andrada, Bryce Grohol, Serrat Nong, Yingguang Liu

**Affiliations:** 1 Medical School, Liberty University College of Osteopathic Medicine, Lynchburg, USA; 2 Oncology, Liberty University College of Osteopathic Medicine, Lynchburg, USA; 3 Molecular and Cellular Sciences, Liberty University College of Osteopathic Medicine, Lynchburg, USA

**Keywords:** bt-20 cells, anti-cancer therapy, 4t1 cells, antiretroviral agent, metastasis, dolutegravir, mouse mammary tumor virus, herv-k, breast cancer, endogenous retroviruses

## Abstract

Increasing evidence points to the role of endogenous retroviruses (ERVs) in driving cancer cell proliferation. The purpose of this study was to explore the possibility of repurposing antiretroviral agents to inhibit ERVs as a new approach to cancer treatment. We found that an integrase strand-transfer inhibitor, dolutegravir (DTG), effectively inhibited the proliferation of multiple cancer cell lines and its antiproliferative potency was positively correlated with the expression levels of the human endogenous retrovirus type K (HERV-K). DTG inhibited the expression of HERV-K in multiple human cancer cell lines and the mouse mammary tumor virus (MMTV) in the murine 4T1 mammary cancer cell line. We chose the fast-growing BT-20 cell line as a model to study the in vitro antiproliferative mechanisms of DTG. BT-20 cells overexpressing both HERV-K *env* and *pol* genes became more resistant to DTG than cells transduced with vector alone. Knockdown of HERV-K also increased DTG resistance of BT-20 cells. The antiproliferative effect of DTG correlated with enhanced expression of E-cadherin and reduction in cell motility and invasiveness. Surprisingly, DTG stimulated expression of the *env* gene of MMTV in vivo and promoted metastasis of 4T1 tumor cells to the lungs. Taken together, our data support the role of ERVs in tumor development and encourage the further search for antiretroviral agents to treat malignancies in which ERVs are active.

## Introduction

Endogenous retroviruses (ERVs) are repetitive DNA sequences in eukaryotic genomes that resemble the DNA genomes of retroviruses. ERVs constitute significant portions of mammalian genomes. Unlike retroviruses that are acquired through horizontal transmission between individual organisms, ERVs are transmitted vertically in the Mendelian fashion. ERVs may play important roles in embryonic development [1], and like other genes involved in early development, ERV genes are actively expressed in many malignancies [2]. Moreover, the Env protein of one group of human ERV, HERV-K, has been demonstrated to induce cell proliferation, migration, invasion, tumor formation, and epithelial-mesenchymal transition (EMT) through the ERK1/2 pathway [3,4]. In addition, the long terminal repeat of HERV-K is involved in progesterone-driven breast cancer cell proliferation [5]. Consequently, there have been explorations to target ERVs as a novel approach to cancer therapy [6,7]. Antiretroviral agents used to treat HIV/AIDS including reverse transcriptase inhibitors (RTI), protease inhibitors (PI), and integrase strand transfer inhibitors (INSTIs) have been found effective in inhibiting HERV-K replication [8]. On the other hand, some antiretroviral drugs have been shown effective in suppressing tumor cell growth in vitro and in vivo [6,7,9]. However, the antineoplastic effect of these agents has not been attributed to their antiviral effects. For example, efavirenz, a non-nucleoside RTI, is found to target the reverse transcriptase of L1 elements, and this is thought to be the chief antineoplastic mechanism [10]. Nelfinavir, a PI, has been found to exert its antineoplastic effect through the generation of reactive oxygen species (ROS) [11].

RTIs are not good tools to investigate the relationship between the antiretroviral effect and the antineoplastic effect because long interspersed nuclear elements (LINEs) also carry reverse transcriptase whose activation is a hallmark of cancer. On the other hand, PIs and INSTIs are more specific inhibitors of ERVs. Raltegravir (INSTI), darunavir (PI), and lopinavir (PI) have all been found to inhibit HERV-K [8]. In the study reported below, we found that dolutegravir (DTG), an INSTI that has been used to inhibit HERV-K in patients with amyotrophic lateral sclerosis [12], has potent antiproliferative effects in cancer cells where HERV-K is active. Using overexpression and knockdown cell lines, we demonstrated that DTG exerts antiproliferative and antimigration effects through inhibition of HERV-K, although the antiretroviral effect is not the only cytotoxic mechanism. We also tested the potential effect of DTG on tumor growth and metastasis in a mouse mammary cancer model where the mouse mammary tumor virus (MMTV, a homolog of HERV-K) plays a tumorigenic role [13].

Portions of this article were previously presented as a meeting abstract at the 2021 Annual Meeting of the American Association for Cancer Research in April 2021. 

This article was previously posted to the Preprints server on February 16, 2022.

## Materials and methods

Cell lines, culturing, and in vitro drug treatment

BT-20 breast tumor cell line (kindly provided by Dr. Jiayuh Lin of the University of Maryland) was maintained in Eagle's minimum essential medium (EMEM) with 10% fetal bovine serum (FBS) and 5% CO_2_. T47D breast cancer cell line (kindly provided by Dr. Joseph Brewer of Edward Via College of Osteopathic Medicine) and 4T1 murine mammary cancer cell line (American type culture collection, ATCC, Manassas, Virginia, USA) were maintained in RPMI 1640 with 10% FBS and 5% CO_2_. MBA-MD-453 breast cancer cell line (kindly provided by Dr. Jiayuh Lin, University of Maryland) was maintained in Leibovitz's L-15 medium in atmospheric air. SKBR3 breast cancer cell line (kindly provided by Dr. Jiayuh Lin, University of Maryland), LNCaP prostate cancer cell line (ATCC), and HEK293T cells (ATCC) were maintained in Dulbecco's modified Eagle medium (DMEM) with 10% FBS and 5% CO_2_. All cells were cultured at 37˚C. DTG (Advanced ChemBlocks, Hayward, California, USA) was dissolved in dimethylsulfoxide (DMSO) and added to culture media in concentrations between 10 μM and 100 μM depending on the susceptibility of the cell lines. Cells were treated for 3-5 days in the presence of the drug, depending on the cell line. For induction of ERV genes in overexpressing cell lines, doxycycline (Cayman Chemical, Ann Arbor, Michigan, USA) was dissolved in DMSO and added to culture media at a final concentration of 0.1 mg/mL. The same volume of DMSO solvent was added to the control cells. After incubation, cells were trypsinized and counted using a NucleoCounter-3000 (ChemoMetec, Allerod, Denmark) according to the manufacturer’s instructions. Most counting was done with the Viability and Cell Count Assay, except for T47D cells which were counted with the Aggregated Cell Count Assay.

Extraction of RNA, reverse transcription, and quantitative PCR

Total cell RNA was extracted from cultured cells or homogenized tissues using the RNeasy Plus Mini Kit (Qiagen, Venio, Netherlands). Complementary DNA was prepared using the Quantitect Reverse Transcription Kit (Qiagen). Real-time polymerase chain reaction (PCR) was performed using the LightCycler 96 (Roche, Basel, Switzerland) and SsoAdvanced Universal SYBR Green Supermix (Bio-Rad, Hercules, California, USA) or AzuraQuant Green Fast qPCR Mix (Real Time Primers, Elkins Park, Pennsylvania, USA). A pair of primers targeted the *pol *gene of HERV-K107 and HERV-K108 (see Table 1 for qPCR primers). Another pair of primers targeted the *env* gene of HERV-K108. The *YWHAZ* gene encoding the 14-3-3 protein zeta/delta was used as the reference gene (for primer sequences, see [14]) for quantification of HERV-K transcripts. The* GAPDH* gene was used as a control for the quantification of genomic HERV-K. For quantification of MMTV transcripts, the mouse gene for phosphoglycerate kinase 1 (*Pgk1*) was used as the reference. Primers of* Pgk1, YWHAZ*, and *GAPDH* were used at 100 nM. Primers for HERV-K and MMTV genes were used at 400 nM. HERV-K genes and the human *YWHAZ* gene were amplified at 95°C 15”, 57°C 25”, and 72°C 45”, while MMTV genes and the mouse *Pgk1* gene were amplified at 95°C 15”, 58°C 25”, and 72°C 45”. 

**Table 1:** Primers for a quantitative polymerase chain reaction

Gene	Forward	Reverse
HERV-K *pol*	CACTCAAGAGGCAGGAGTTAAT	GGCCTGTCCTTGGGAATTAT
HERV-K *env*	ATTGGCAACACCGTATTCTGCT	CAGTCAAAATATGGACGGATGGC
MMTV *env*	TTAGTTAAGGAGATGCAAACTGC	CACATCTTGTCCCAACTCTAAAAC
MMTV *pol*	GACCAGCCTGTATGGCTTAAT	GAGGAGCGAGCAGGTGAACT
Mouse *Pgk1*	GCAGATTGTTTGGAATGGTC	TGCTCACATGGCTGACTTTA
*GAPDH*	AACTTTGGCATTGTGGAAGG	GCAGGGATGATGTTCTGG
YWHAZ	ACTTTTGGTACATTGTGGCTTCAA	CCGCCAGGACAAACCAGTAT

For quantitation of HERV-K genes in genomic DNA, the Quick-DNA Miniprep Kit (Zymo Research, Irvine, California, USA) was used to extract the DNA. Primers for* env* and *pol* were the same as in the reverse-transcription PCR (RT-PCR) described above. The reference gene was also *YWHAZ*.

Plasmids and constructs

The *env* and *pol* genes of HERV-K were amplified from BAC clones RP11-33P21 (containing the HERV-K108 sequence) and CTB-69E10 (containing the HERV-K107 *gag-pro-pol* gene) (see Table 2 for primer sequences). The BAC clones were purchased from ThermoFisher (Waltham, Massachusetts, USA). The *env* and *pol* genes of MMTV were amplified from mouse genomic DNA extracted from the 4T1 cell line. CloneAmp HiFi PCR Premix (Takara Bio, San Jose, California, USA) was used to amplify the target genes. The lentiviral vector, pLenti-puro, was a gift from Le-Ming Shih (Addgene plasmid # 39481 ; http://n2t.net/addgene:39481 ; RRID:Addgene_39481) [15]. It was digested with BstBI and BamHI for cloning of HERV-K genes. In-Fusion® HD Cloning Plus Kit or In-Fusion Snap Assembly Master Mix (Takara Bio) was used in making the overexpression constructs. For HERV-K knockdown, the ERVK-6 Human shRNA Plasmid Kit was purchased from OriGene (Rockville, Maryland, USA).

**Table 2:** Primers for cloning

Gene	Forward	Reverse	Reference sequence
HERV-K *pol*	GTCGACTAGTGGATCTCGGAAGAAGCTAGGGTGATAATGG	ATAGGCTTACCTTCGTCCTGGTGAAACACAAGCAAAACC	1091-6411 of AF164613.1
HERV-K *env*	GTCGACTAGTGGATCAGGGAAGGTGATAACGTGGGG	ATAGGCTTACCTTCGCATGTTTCAGAGAGCACGGGG	30253-32563 of AC072054.10
MMTV *pol*	GAGATCGTCGACTAGGCAGTCTCGCCTACAGAGAAG	GATGACCGGTACGCGAGAGGTTTGGGGAGTTTGTGA	266-5603 of NC_001503.1
MMTV *env*	CAGTGTGGTGGAATTGGACGAGGCTATGCTTGTGTT	GCCCTCTAGACTCGACGTCCTTGGTGGGAAACAAC	5333-7468 of NC_001503.1

Lentiviral packaging and transduction

Overexpression constructs or shRNA knockdown plasmids were used to transfect HEK293 cells along with the Env-expressing plasmid pCMV-VSV-G and the packaging plasmid pCMV-dR8.2 dvpr in an equimolar ratio. Both pCMV-VSV-G and pCMV-dR8.2 dvpr were gifts from Bob Weinberg (Addgene plasmid # 8454 and 8455; http://n2t.net/addgene:8454 and http://n2t.net/addgene:8455; RRID: Addgene_8454 and Addgene_8455). The Lipofectamine 3000 reagent and OptiMEM I reduced serum medium (both from ThermoFisher) were used in the transfection protocols according to the manufacturer’s instructions. Culture supernatant was used to transduce target cells in the presence of polybrene at 8 μg/mL. For the selection of transduced cells, puromycin concentration was gradually increased from 0.5 μg/mL to 1 μg/mL for both BT-20 and T47D cell lines.

Immunoblotting

Cells were lysed with RIPA buffer containing the mammalian PI cocktail (Genesee Scientific, San Diego, California, USA) and electrophorized in sodium dodecyl sulfate-polyacrylamide gels. Samples were transferred onto a nitrocellulose membrane. Rabbit polyclonal anti-ERK-9 Env (MyBioSource, San Diego, California, USA) and mouse monoclonal anti-HERVK capsid (Austral Biologicals, San Ramon, California, USA) were used at 1:5000. Mouse monoclonal anti-E-Cadherin (American Research Products, Waltham, Massachusetts, USA) was used at 1:20. Reaction with the above three primary antibodies was carried out at 4°C overnight. Mouse monoclonal anti-V5 Tag (ThermoFisher) was used at 1:5000 and incubated at room temperature for 1.5 hours. Horseradish peroxidase (HRP)-conjugated mouse monoclonal anti-GAPDH (MyBioSource) was used at 1:40,000 and incubated at room temperature for 1 hour. HRP-conjugated goat anti-mouse IgG and HRP-conjugated goat anti-rabbit IgG (both from Boster Bio, Pleasanton, California, USA) were used at 1:20,000. Reaction with the above two secondary antibodies was carried out at room temperature for 1-1.5 hours. Enhanced chemiluminescence (Boster Bio) was photographed using the ChemiDoc XRS+ System (Bio-Rad).

ROS assay

Intracellular ROS was quantified using the DCFDA/H2DCFDA-Cellular ROS Assay Kit (Abcam, Cambridge, UK) according to the manufacturer’s instructions. T47D cells were cultured in RPMI1640 medium without phenol red and seeded onto a clear-bottom 96-well microplate at 25,000 cells per well. The final concentration of DTG was 20 μM and the final concentration of nelfinavir (Sigma-Aldrich, Burlington, Massachusetts) was 10 μM. Samples were in triplicates. Fluorescence was read at Ex/Em = 485/520 nm in endpoint mode using the FLUOstar® Omega microplate reader (BMG LABTECH, Ortenberg, Germany).

Cell cycle analysis

The NucleoCounter-3000 (ChemoMetec) was used to perform the DAPI-based two-step cell cycle assay according to the manufacturer’s instructions.

Wound-healing assay

BT-20 cells were pre-treated with 50 μM DTG or DMSO for 48 hours and seeded in quadruplet wells on a 24-well plate in serum-free EMEM. DTG-treated cells continued to receive the same concentration of drug during wound healing. After allowing 4 hours for attachment, cells were scratched and photographed at 4x for 22 hours at 30-minute intervals using a BZ-X800 microscope (Keyence, Osaka, Japan) while the cells are incubated at 37°C with 5% CO_2_. Relative wound density was calculated according to Grada et al. [16].

Transwell invasion assay

Transwell invasion assay was performed using the Cultrex Basement Membrane Extract Cell Invasion Assay Kit (R&D Systems, Minneapolis, Minnesota, USA). Top wells were treated with 0.1x or 0.5x basement membrane extract. BT-20 cells were pretreated with 50 μM DTG or DMSO solvent for 24 hours and seeded in top wells of a 96-well plate at 3 x 10^4^ cells per well in DMEM with 0.5% FBS. DTG-treated cells were continuously treated with the same concentration of drug during the assay. After incubation for 65 hours, cells in the top wells were trypsinized and counted using the NucleoCounter-3000, while cells in the bottom wells were stained with crystal violet and counted using the Image Cytometry function of the BZ-X800 microscope (Keyence).

Inoculation and treatment of mice

BALB/c mice 7-8 weeks old were inoculated with 15,000 4T1 cells suspended in 50 μL of Versene solution (ThermoFisher) subcutaneously using a 30½-gauge needle into the fourth mammary gland. Treatment with DTG was administered on the same day and every day for 32 days. The drug was suspended in phosphate-buffered saline (PBS) and administrated at 10 mg/kg/day by subcutaneous injection on the back. Control mice received the same volume of PBS. Mice were weighed twice a week. The palpable tumor usually developed in a week. Thirteen mice were used in the control group and 12 were included in the DTG treatment group.

Metastatic colony count

Thirty-three days after inoculation of 4T1 cells, mice were sacrificed. Lungs, the most common site of metastasis [17], were collected aseptically, minced, and digested with 5 mL of PBS containing collagenase type IV (ThermoFisher) at 1 mg/mL and elastase (Sigma-Aldrich) at 6 U/mL for 1 hour at 4°C. The suspension was filtered through a 70 μM PET-mesh strainer (PluriSelect, Leipzig, Sachsen, Germany). Cells were collected by centrifugation at 125 g for 8 minutes, washed with 10 mL of PBS, and resuspended in 10 mL of RPMI1640 with 10% FBS, 6-thioguanine (Cayman Chemical) at 10 μg/mL, as well as Antibiotic-Antimycotic (Gibco, Jenks, Oklahoma) consisting of penicillin at 100 unit/mL, streptomycin at 100 μg/mL, and amphotericin B at 0.25 μg/mL. Three 10-fold dilutions were made in 100-mm Petri dishes. After incubation at 37°C with 5% CO_2_ for 13 days, colonies were stained with crystal violet and counted. 

Statistical analysis

The Mann-Whitney U test was used for quantitative data. Statistical significance was called at p <0.05. Correlation coefficients were tested using the Social Science Statistics Calculator [18].

## Results

DTG inhibited HERV-K and MMTV in cancer cells

**Figure 1:**
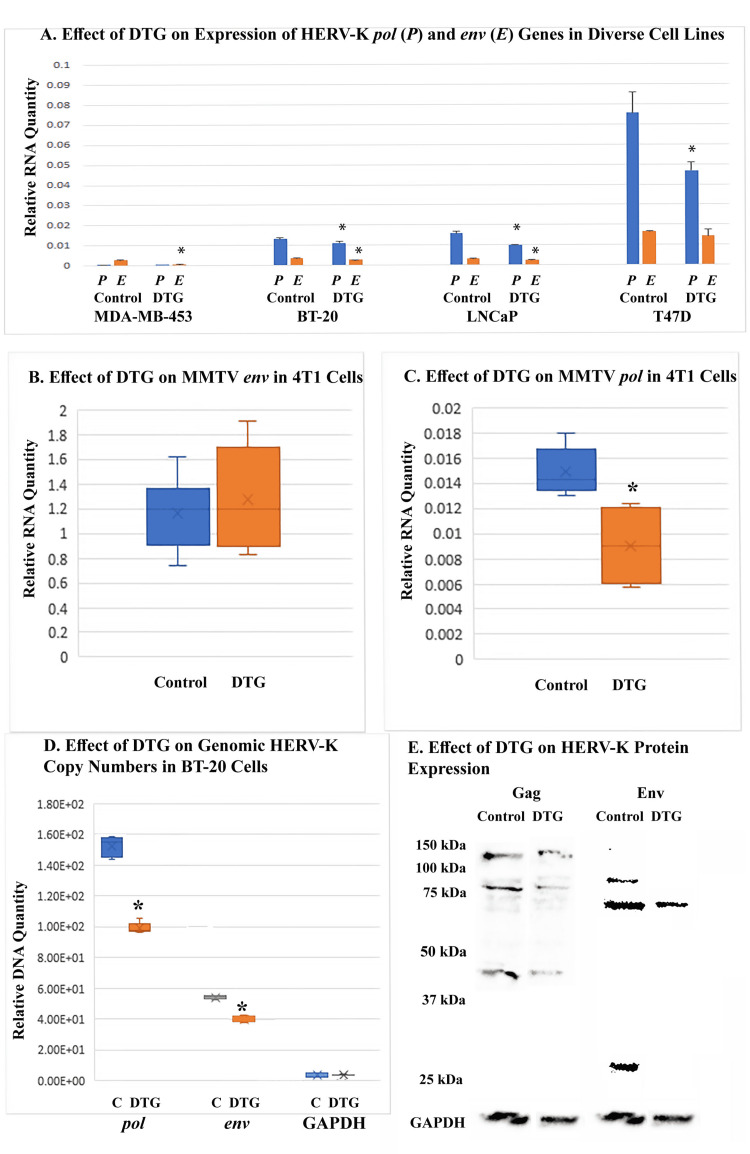
Effect of DTG on expression and replication of endogenous retroviruses (A) shows representative results of quantitative RT-PCR on the expression of HERV-K genes in various cell lines. Cells were treated with DTG at 100 μM (MDA-MB-453 cells), 50 μM (LNCaP cells and BT-20 cells), and 20 μM (T47D cells) for 3-4 days depending on the proliferation rates of the cell lines. Control cells received DMSO solvent. Error bars indicate standard deviations of three technical replicates. Asterisks denote statistically significant differences from solvent-treated control cells.  (B) and (C) show pooled data of quantitative RT-PCR on the expression of MMTV genes in 4T1 cells using two independent experiments, each with three technical replicates. Cells were treated with DTG at 20 μM or DMSO solvent for four days. Asterisk denotes a statistically significant difference from solvent-treated control. (D) shows the relative abundance of HERV-K proviral DNA in the genome of BT-20 cells as measured with qPCR. Cells were treated with DTG at 50 μM for two days and 75 μM for two more days. DNA quantities were normalized against that of *YWHAZ*. The *GAPDH* gene was used as a negative control. Each treatment included two biological samples, and each sample was quantified in three technical replicates. Asterisks denote statistically significant differences from solvent-treated C cells. (E) shows Western blot analysis of HERV-K Gag and Env proteins upon DTG treatment of BT-20 cells. Cells were treated with DTG at 100 μM or DMSO solvent (control) for three days. DTG, dolutegravir; RT-PCR, reverse-transcription polymerase chain reaction.

Quantitative RT-PCR revealed that DTG inhibited both the *pol* and *env* genes of HERV-K in multiple cell lines including the breast cancer cell lines MDA-MB-453, BT-20, and T47D, as well as the LNCaP prostate cancer cell line (Figure 1A). The *pol* gene of HERV-K was more significantly inhibited than the *env* gene. DTG also inhibited the expression of the *pol *gene, but not the *env* gene, of MMTV in the murine 4T1 mammary cancer cell line (Figures 1B, 1C). Moreover, DTG treatment of BT-20 cells reduced the quantity of HERV-K genes in genomic DNA (Figure 1D), while the quantity of the *GAPDH* (glyceraldehyde-3-phosphate dehydrogenase) gene in genomic DNA was not affected. Western blot analysis revealed inhibition of the Gag and Env proteins of HERV-K in BT-20 cells by DTG (Figure 1E). Using a monoclonal anti-Gag, we were able to detect in BT-20 cells a 120-kDa (presumably Gag-Pro-Pol) polyprotein, 80-kDa (presumably Gag-Pro) polyprotein, a 44-kDa intermediate product, a 34-kDa product, and a 30-kDa product. The blot in Figure 1E shows the expression of the three larger proteins being inhibited by DTG. Using a polyclonal anti-Env, we detected in BT-20 cells three Env bands of 90 kDa, 68 kDa, and 27 kDa. All three Env-related products were downregulated by DTG.

Antiproliferative effect of DTG was correlated with HERV-K expression

**Figure 2:**
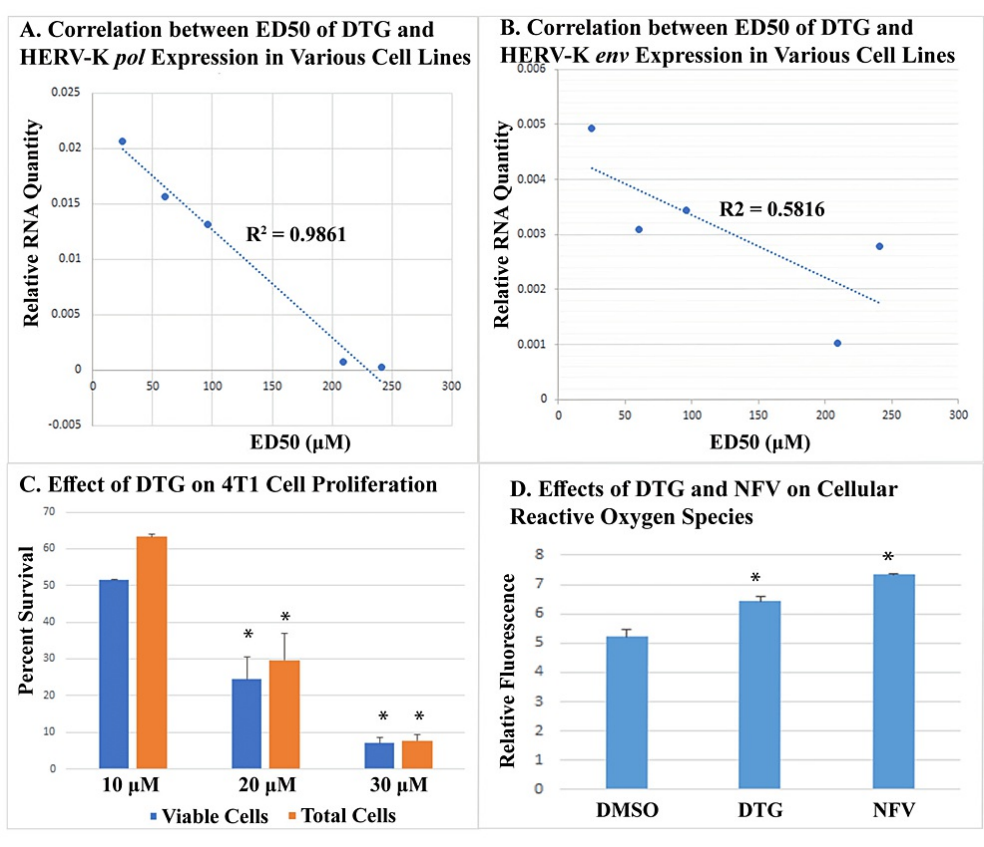
Effect of DTG on cancer cell proliferation (A) and (B) show the correlation between HERV-K expression and ED50 of DTG in five human cancer cell lines (T47D, LNCaP, BT-20, SKBR3, and MDA-B-453 from low to high ED50). Cells were treated with DTG for 3-4 days. ED50 was calculated using the Quest Graph™  ED50 Calculator (https://www.aatbio.com/tools/ed50-calculator). Expressions of HERV-K genes were measured using quantitative RT-PCR. (C) shows the effect of DTG on the proliferation of mouse 4T1 cells. Cells were treated with DTG for four days. Error bars indicate standard deviations of biological duplicates. Asterisks denote significant differences from cells treated with lower concentrations of DTG. (D) shows the effect of DTG and NFV on the production of reactive oxygen species. Relative fluorescence indicates the amount of cellular reactive oxygen species. Asterisks denote a significant difference from DMSO-treated control cells. DTG, dolutegravir; RT-PCR, reverse transcription polymerase chain reaction; NFV, nelfinavir; DMSO, dimethyl sulfoxide.

DTG inhibited the proliferation of multiple cancer cell lines. The ED50 varied from over 200 μM in MDA-MB-453 cells and SKBR3 cells to under 20 μM in mouse 4T1 cells. In the five human breast and prostate cancer cell lines tested (MDA-MB-453, SKBR3, BT-20, LNCaP, and T47D), the antiproliferative effect of DTG was strongly correlated with the expression of HERV-K in the cell lines. The correlation coefficient between ED50 of DTG and the expression of HERV-K *pol* was 0.99, and that between ED50 and the expression of HERV-K *env* was 0.76, with the former being statistically significant (Figures 2A, 2B). Figure 2C shows the dosage-dependent antiproliferative effect of DTG on 4T1 cells.

While the correlation between ED50 and HERV-K expression pointed to HERV-K being the mediator of the antiproliferative efficacy of DTG, we found that the drug also induced the production of ROS in the T47D breast cancer cells (Figure 2D). Nelfinavir, a known inducer of ROS, was used as a positive control in the ROS assay.

Overexpressing HERV-K increased the resistance of BT-20 cells against DTG

**Figure 3:**
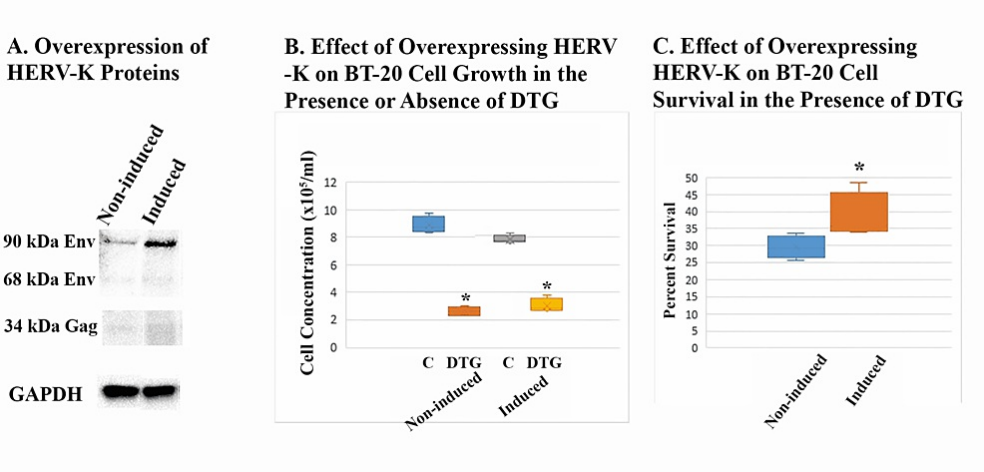
Effect of overexpressing HERV-K on DTG resistance of BT-20 cells (A) shows Western blot analysis of Env and Gag proteins of HERV-K in overexpressing cells. BT-20 cells transduced with both *env *and *pol* genes of HERV-K under a tet operator were cultured in the absence (non-induced) or presence (induced) of 0.1 μg/mL doxycycline for five days. Transduced Env protein was detected using anti-V5 Tag, while Gag was detected using a specific anti-Gag. (B) shows the effect of overexpressing both *env* and *pol* on the growth of BT-20 cells in the absence or presence of DTG as compared to non-induced cells. Non-induced cells were treated with DTG or DMSO solvent (C denotes solvent control) for four days, while induced cells were pretreated with 0.1 μg/mL doxycycline for 24 hours before a four-day treatment with DTG + doxycycline or solvent + doxycycline. Viable cells were counted in quadruplicate samples. Asterisks denote statistically significant differences from C cells. (C) shows the effect of overexpressing HERV-K genes on resistance of BT-20 to DTG. Percent survival was calculated by dividing the number of viable cells in DTG-treated samples by the average number of cells in solvent-treated C samples. Samples were in quadruplets. Asterisk denotes a statistically significant difference from non-induced cells. DTG, dolutegravir; DMSO, dimethyl sulfoxide.

The entire *env* region of HERV-K108, including 98 bases upstream and 112 bases downstream, was cloned into the pLenti-puro vector which expresses target genes under a tet operator. The entire *gag-pro-pol* region of HERV-K107 including 21 bases upstream and 30 bases downstream was also cloned into the same vector. The constructs were packaged in lentiviral particles, transduced into BT-20 cells, and induced with doxycycline to express HERV-K genes (Figure 3A). Overexpression of the HERV-K genes did not measurably alter the proliferation rate of BT-20 cells (Figure 3B). A cell line stably overexpressing both the *env* and *pol* genes demonstrated significantly enhanced resistance against DTG in the presence of doxycycline (Figures 3B, 3C). Cell lines transduced with the empty vector, the *env* gene alone, or the *pol *gene alone did not show increased resistance to DTG in the presence of doxycycline (not shown).

Knocking down HERV-K also increased the resistance of BT-20 and T47D cells against DTG

**Figure 4:**
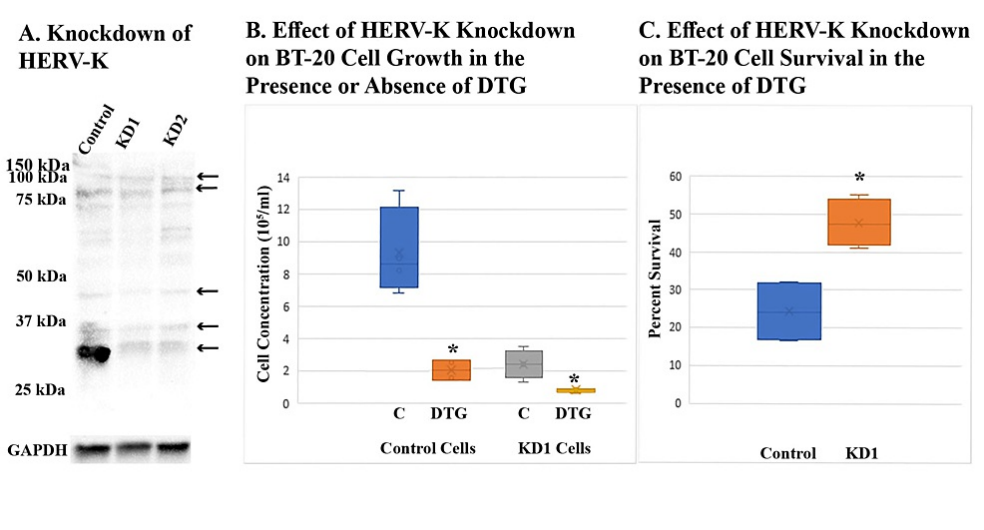
Effect of HERV-K knockdown on DTG resistance of BT-20 cells (A) shows Western blot analysis of HERV-K expression in two knockdown cell lines, KD1 and KD2, using an anti-Gag. Control cells were transduced with the same vector encoding random shRNA. Arrows point to Gag bands. (B) shows the effect of HERV-K knockdown on the growth of BT-20 cells in the absence or presence of DTG as compared to the control cell line. C denotes cells treated with DMSO solvent only. DTG treatment lasted five days at 100 μM and two additional days at 50 μM. Viable cells were counted in quadruplicate samples. Asterisks denote statistically significant differences from C cells. (C) shows the effect of HERV-K knockdown on resistance of BT-20 cells to DTG. Percent survival was calculated by dividing the number of viable cells in DTG-treated samples by the average number of cells in solvent-treated C samples. Samples were in quadruplets. Asterisk denotes a statistically significant difference from control cells. DTG, dolutegravir; DMSO, dimethyl sulfoxide.

**Figure 5:**
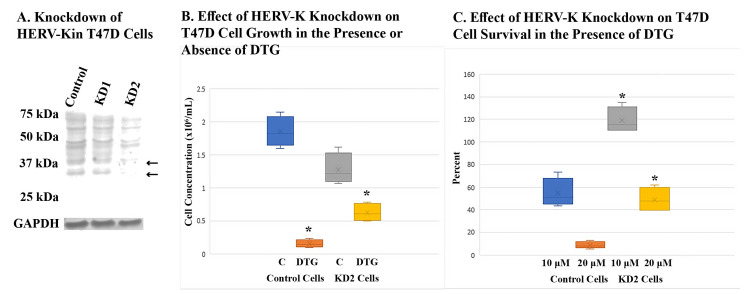
Effect of HERV-K knockdown on DTG resistance of T47D cells (A) shows Western blot analysis of HERV-K expression in two knockdown cell lines, KD1 and KD2, using an anti-Gag. Control cells were transduced with the same vector encoding random shRNA. Arrows point to Gag proteins that were knocked down. (B) shows the effect of HERV-K knockdown on the growth of T47D cells in the absence or presence of DTG as compared to the control cell line. C denotes cells treated with DMSO solvent. DTG treatment lasted five days at 20 μM. Total cells were counted in quadruplicate samples. Asterisks denote statistically significant differences from C cells. (C) shows the effect of HERV-K knockdown on resistance of T47D cells to DTG at two drug concentrations. Percent survival was calculated by dividing the number of total cells in DTG-treated samples by the average number of cells in solvent-treated C samples. Samples were in quadruplets. Asterisks denote statistically significant differences from control cells treated with DTG at the same concentration. DTG, dolutegravir; DMSO, dimethyl sulfoxide.

Two HERV-K knockdown lines of BT-20 cells were established, one with shRNA targeting the *pol* region (6175-6203 of the HERV-K108 genome, KD1), and one with shRNA targeting the *pro *region (3327-3355 of the HERV-K108 genome, KD2). BT-20 cells transduced with random shRNA served as a control. An anti-Gag antibody revealed reduced expression of the 30-kDa Gag product in the knockdown cell lines (Figure 4A). The knockdown cell lines proliferated slower and became more resistant to DTG. Figures 4B, 4C show the proliferation and survival of one of the knockdown cell lines. Knockdown T47D cell lines were also established with the same shRNAs. T47D cells yielded a different pattern of Gag bands upon immunoblotting (Figure 5A). Like in BT-20 cells, the smaller products (37 kDa and 30 kDa) were more sensitive to knockdown. Figures 5B, 5C show slower proliferation and increased DTG resistance of one of the knockdown T47D cell lines. 

The effect of DTG on the cell cycle of BT-20 cells was similar to HERV-K knockdown

**Figure 6:**
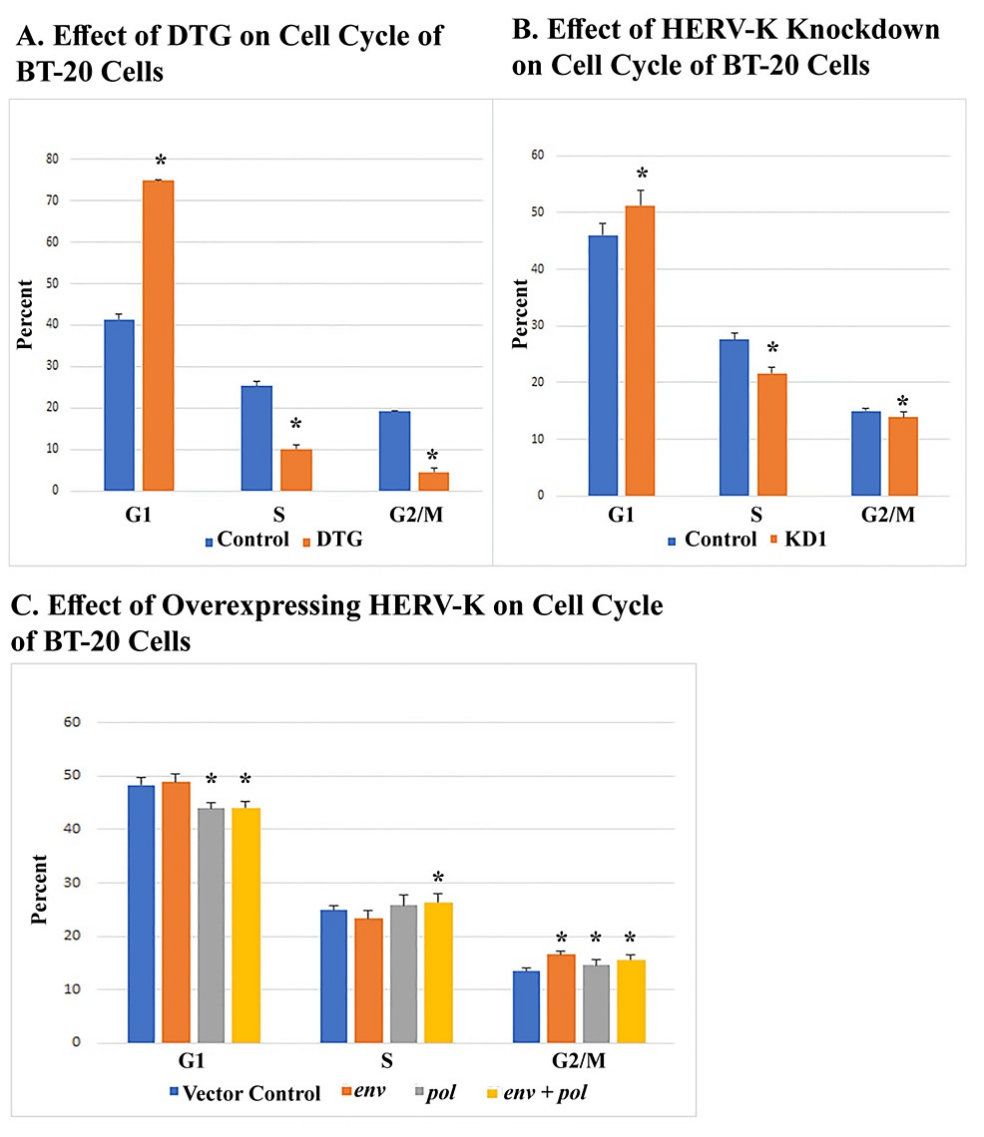
Effect of DTG and HERV-K on BT-20 cell cycle (A) shows the effect of treatment with DTG at 50 μM for 26 hours. Control cells were treated with DMSO solvent. (B) shows the effect of HERV-K knockdown. Control cells were transduced with the same vector plasmid expressing random shRNA. (C) shows the effect of overexpressing *env* alone, *pol* alone, or both. Control cells were transduced with vector only. All cells were incubated in the presence of 0.1 μg/mL doxycycline for 24 hours. All asterisks denote statistically significant differences from control. DTG, dolutegravir; DMSO, dimethyl sulfoxide.

Cell cycle analyses revealed that DTG kept more cells in the G1 phase and reduced the number of cells in the S, G2, and M phases (Figure 6A). HERV-K knockdown resulted in the same pattern of changes (Figure 6B). Cells overexpressing *pol* alone or *pol + env* showed decreased number of G1 cells and an increased number of S, G2, and M cells, the exact opposite of DTG or HERV-K knockdown, and the effects were more prominent in doubly transduced cells (Figure 6C). BT-20 cells overexpressing the *env *gene alone also showed an increased number of G2/M cells, although there was no change in the measured number of G1 cells. Env-driven increase of G2/M cells seemed to be chiefly due to a shortened S phase (Figure 6C).

DTG and HERV-K knockdown both inhibited cell motility and invasiveness

**Figure 7:**
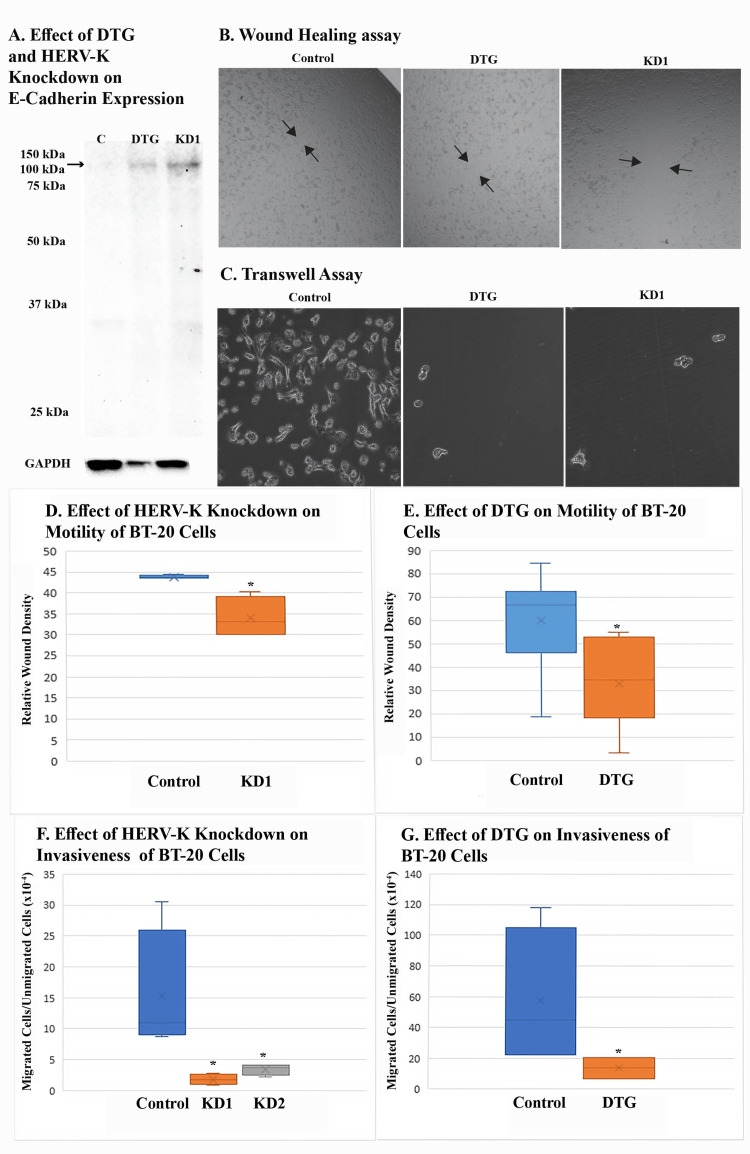
Effect of DTG on the motility and invasiveness of BT-20 cells as compared with HERV-K knockdown (A) shows Western blot analysis of E-Cadherin expression (arrow) in DTG-treated or knockdown cells. Cells transduced with random shRNA were treated with solvent only (C) or DTG at 50 μM for five days. KD1 cells were treated with DMSO solvent. A monoclonal anti-E-Cadherin was used. (B) Wound-healing assay. Control cells were transduced with vector plasmid encoding random shRNA. DTG cells were pretreated with 50 μM DTG for 48 hours and the treatment continued during wound healing. Images represent the wounded area after 22 hours. (C) Transwell assay. DTG cells were pretreated with 50 μM DTG or solvent for 24 hours and the treatment continued during the assay. Images were taken after 65 hours. (D) and (E) Results of the wound-healing assay in terms of relative wound density. Control cells in D were transduced with plasmid encoding random shRNA while control cells in E were treated with DMSO solvent. (F) and (G) Results of transwell assay in terms of the ratio of migrated cells to unmigrated cells. All asterisks denote statistically significant differences from control. DTG, dolutegravir; DMSO, dimethyl sulfoxide.

DTG and HERV-K knockdown both increased expression of E-Cadherin in BT-20 cells (Figure 7A). Since the loss of E-Cadherin expression has been associated with EMT and metastasis, we measured the motility of BT-20 cells in the presence of DTG using a wound-healing assay. As expected, DTG treatment reduced the motility of BT-20 cells, so did HERV-K knockdown (Figures 7B, 7D, 7E). A transwell assay revealed reduced invasiveness of BT-20 cells through the basement membrane extract in the presence of DTG, an effect similar to that of HERV-K knockdown (Figures 7C, 7F, 6G).

DTG enhanced HERV-K *env* expression and metastasis of 4T1 cells in vivo

**Figure 8:**
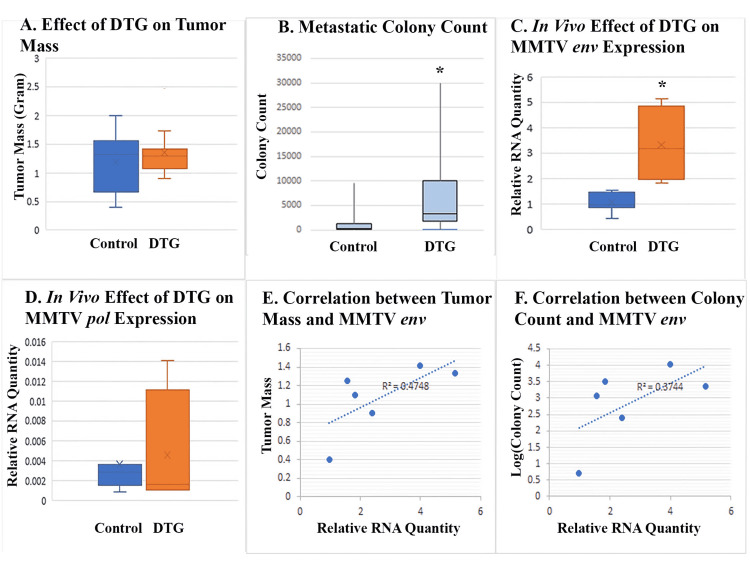
In vivo effect of DTG on tumor growth and MMTV expression (A) shows average tumor mass in DTG-treated mice versus solvent-treated mice. (B) shows the number of metastatic cells in the lungs of DTG-treated mice versus solvent-treated mice. Metastatic cells were represented as thioguanine-resistant colonies grown from digested lungs. (C) and (D) show the effect of DTG on MMTV expression in tumor tissues as measured with quantitative RT-PCR. (E) and (F) show the correlation between MMTV* env* expression in tumor tissue and tumor mass or metastatic colony count. Asterisks denote statistically significant differences from control. DTG, dolutegravir; RT-PCR, reverse-transcription polymerase chain reaction; MMTV, mouse mammary tumor virus.

In BALB/c mice inoculated with 4T1 cells orthotopically, treatment with DTG did not affect the size of mammary tumors (Figure 8A). Surprisingly, DTG increased the number of metastatic cells in the lungs (Figure 8B), but this was associated with increased expression of MMTV *env* in tumor tissues of DTG-treated mice (Figure 8C) while the effect of DTG on MMTV *pol* in tumor tissues was not significant (Figure 8D). Levels of *env* gene expression in tumor tissues were positively correlated with both tumor mass and metastatic colony counts (Figures 8E, 8F).

## Discussion

Although there have been efforts to use antiretroviral agents to inhibit cancer cell proliferation in vitro and to suppress cancer growth in vivo [6,7,9,11], this study is the first documented attempt to prove that an antiretroviral agent, DTG, exerts its antineoplastic effect through inhibition of an ERV. Overexpressing HERV-K genes bypasses the inhibitory effect of DTG on HERV-K replication, thus reducing the potency of the drug on cell proliferation. On the other hand, knocking down HERV-K eliminates the target of DTG, also rendering cells resistant to the drug. DTG has been reported to increase intracellular calcium levels and induce oxidative stress in erythrocytes [19]. Our finding of increased ROS in T47D breast cancer cells suggests this mechanism as well. A DTG derivative is known to inhibit the proliferation of non-small-cell lung cancer cells through the calcium signaling pathway [20]. In agreement with these findings suggesting other mechanisms, our data indicate that ERV inhibition is not the only antineoplastic mechanism of DTG, since neither overexpressing HERV-K genes nor knockdown of HERV-K renders BT-20 cells completely resistant to DTG.

Because HERVs are mostly degenerate and not actively transposing in the human genome, the inhibitory effect of INSTIs on HERV-K expression [8,12] is puzzling. Our finding of the effect of DTG on HERV-K quantity in genomic DNA indicates dynamic changes of HERV-K on the DNA level in BT-20 cells. Since the copy number of HERV-W is known to increase in peripheral blood mononuclear cells of patients with multiple sclerosis [21], it is conceivable for the more intact HERV-K to be actively transposing in malignant cancers.

The integrase plays other roles in retroviral replication besides the integration of proviral DNA into host chromosomes. Cleavage of episomal 2-LTR circles and connection of episomal proviral DNA into linear concatemers are also functions of the integrase [22,23]. These episomal forms of viral DNA are transcribed to various degrees [24]. In addition, the integrase also plays roles in reverse transcription, recruitment of host proteins into the viral particle, as well as viral maturation [25]. Even without new insertion events, INSTIs can affect many aspects of ERV activities in host cells.

Surprisingly, the inhibitory effect of DTG on MMTV *pol* observed in vitro became insignificant in vivo and the drug significantly enhanced *env* expression in mouse tumor tissues, accompanied by enhanced metastasis to the lungs. These results support previous findings of the HERV-K Env in driving cancer cell proliferation and EMT [2,3]. We postulate the following possible mechanisms for the discrepancy between *i*n vitro and in vivo findings concerning the effects of DTG on ERV expression and tumor development.

1. The in vivo ERV-stimulating effect of DTG may have to do with oxidative stress, which would inhibit DNA methylation and histone deacetylation in the host cell, thus de-repressing ERV elements [26]. Immunosuppressive cells such as myeloid-derived suppressor cells produce ROS in the tumor microenvironment [27]. Possible synergy between the oxidative effect of immunosuppressive cells and that of DTG may outweigh the antiviral effect of DTG.

2. DTG may have a negative effect on host immunity by inhibiting ERVs or by the production of ROS. ERVs contribute to interferon responses and dendritic cell activation [28,29], and inhibition of ERVs may affect the host immune response. In addition, oxidative stress impedes immune cell signal transduction [27]. The negative impact of DTG on host immunity may outweigh its antiviral and antitumor effects.

3. The timing of tumor tissue harvest may mask the direct effect of DTG on MMTV expression. Since MMTV antigens are known targets of host immunity [30], the amount of the virus in the tumor tissue reflects the combined effects of the antiviral agent and antiviral immunity. Viral RNA in tumor samples taken at necropsy may not reflect the direct action of DTG on viral replication. Whereas cell cultures were continuously exposed to DTG in in vitro experiments, the mice received the drug by intermittent injections. Even if DTG inhibited MMTV at higher concentrations, viral RNA in tumor tissues collected at necropsy (one day after the last administration) may reflect a rebound as the immune response of the treated mice might be less robust than that of the control mice. 

In this study, we did not address the potential impact of DTG on the immune system of tumor-bearing mice. Examination of the quantities and functions of lymphocytes, phagocytes, and myeloid-derived suppressor cells in the tumor, bone marrow, and spleen will likely be fruitful. Since there are fundamental differences between human and mouse ERVs and between human and mouse immune systems, using xenograft models to study the effect of DTG on HERVs and human breast cancers in immunocompromised mice will eliminate the immunity factor and may yield different results from what we observed using MMTV-related murine cancer in immunocompetent mice.

## Conclusions

DTG inhibited proliferation and migration of multiple human cancer cell lines through inhibition of HERV-K, although the drug also caused oxidative stress in cancer cells. However, DTG enhanced the expression of the MMTV in mouse tumor tissues and promoted metastasis of mouse mammary tumors into the lungs. These results highlight the different effects of the same drug in cell cultures and in an animal model.
